# Fungal Microbiota Composition in Inflammatory Bowel Disease Patients: Characterization in Different Phenotypes and Correlation With Clinical Activity and Disease Course

**DOI:** 10.1093/ibd/izad289

**Published:** 2023-12-16

**Authors:** Ignacio Catalán-Serra, Silje Thorsvik, Vidar Beisvag, Torunn Bruland, David Underhill, Arne Kristian Sandvik, Atle van Beelen Granlund

**Affiliations:** Centre of Molecular Inflammation Research, NTNU-Norwegian University of Science and Technology, Trondheim, Norway; Department of Clinical and Molecular Medicine, Norwegian University of Science and Technology, Trondheim, Norway; Gastroenterology, Department of Medicine, Levanger Hospital, Nord-Trøndelag Hospital Trust, Levanger, Norway; Centre of Molecular Inflammation Research, NTNU-Norwegian University of Science and Technology, Trondheim, Norway; Department of Clinical and Molecular Medicine, Norwegian University of Science and Technology, Trondheim, Norway; Department of Clinical and Molecular Medicine, Norwegian University of Science and Technology, Trondheim, Norway; Department of Clinical and Molecular Medicine, Norwegian University of Science and Technology, Trondheim, Norway; Department of Gastroenterology and Hepatology, Clinic of Medicine, St. Olav’s University Hospital, Trondheim, Norway; Centre of Molecular Inflammation Research, NTNU-Norwegian University of Science and Technology, Trondheim, Norway; Research Division of Immunology, Cedars-Sinai Medical Center, Los Angeles, CA, USA; F. Widjaja Foundation Inflammatory Bowel and Immunobiology Research Institute, Cedars-Sinai Medical Center, Los Angeles, CA, USA; Centre of Molecular Inflammation Research, NTNU-Norwegian University of Science and Technology, Trondheim, Norway; Department of Clinical and Molecular Medicine, Norwegian University of Science and Technology, Trondheim, Norway; Department of Gastroenterology and Hepatology, Clinic of Medicine, St. Olav’s University Hospital, Trondheim, Norway; Centre of Molecular Inflammation Research, NTNU-Norwegian University of Science and Technology, Trondheim, Norway; Department of Clinical and Molecular Medicine, Norwegian University of Science and Technology, Trondheim, Norway; Department of Gastroenterology and Hepatology, Clinic of Medicine, St. Olav’s University Hospital, Trondheim, Norway

**Keywords:** microbiome, fungi, mycobiome, inflammatory bowel disease, microbiology, Crohn's disease, ulcerative colitis, prognosis

## Abstract

**Background:**

There is growing evidence of the role of the mycobiome in inflammatory bowel disease (IBD). Variations within phenotypes and activity and with prognosis have been poorly studied.

**Methods:**

A total of 111 individuals were prospectively enrolled: 89 IBD patients (52 ulcerative colitis and 37 Crohn’s disease [CD]) and 22 healthy individuals. Disease characteristics were collected and a fecal calprotectin >100 μg/mg was considered indicative of activity. A subset of patients was followed for 6 ± 2 years. Disease course was designated as either complicated or uncomplicated based on the need of intensified medication and/or surgery. ITS sequencing was performed targeting the ITS1 region.

**Results:**

We found lower *Ascomycota/Basidiomycota* ratio in IBD. Patients showed a marked increase in *Candida dublinensis* and *Ca albicans* and were depleted of *Aspergillus rubrobrunneus* and *Penicillium brevicompactum* (*P* ≤ .001) *Saccharomyces* was predominant in total colitis and *Penicillium* in proctitis. Several *Penicillium* species were depleted in total colitis vs proctitis. Ileal CD patients were enriched in *Debaromyces hansenii* and depleted of *Ca tropicalis* (*P* ≤ .001). *Ca albicans* was overrepresented in inflammatory (B1) vs fibrostenosing (B2) CD. *Ca dublinensis* was more abundant in active patients and correlated positively with fecal calprotectin and neutrophil gelatinase-associated lipocalin, while *S pastorianus* correlated inversely with activity. *Ca sake* was associated with complicated disease and increased abundance of *Cryptococcus carnescens* with the need for surgery in CD.

**Conclusions:**

This study shows important differences in the mycobiome in IBD and within phenotypes. Selected fungal species were associated with complicated disease and the need of surgery in CD. This work adds to our understanding of the role of fungi in IBD, with potential clinical implications.

**Key Messages**

**What is already known?**

- Alterations of the microbiome are a hallmark of inflammatory bowel disease (IBD).
- Mycobiome dysbiosis has been demonstrated in IBD patients.
- The fungal microbiota (mycobiome) plays a key role in the regulation of intestinal homeostasis, and there is growing evidence on its role in the pathogenesis of IBD.

**What is new here?**

- Our study shows important differences in the fecal mycobiome composition in IBD patients from Norway, and also between ulcerative colitis and Crohn’s disease.
- We make a systematic study of how the gut mycobiome changes within the different phenotypes of the disease and how it correlates with disease activity.
- Our study novel provides information on how these fungal signatures are associated with worse outcomes after follow-up.

**How can this study help patient care?**

- A better characterization of fungi as an essential part of the gut microbiome in IBD patients can lead to a better understanding of the pathogenesis, personalized strategies to predict disease outcomes, and microbiome-based therapies that improve the current therapeutic strategies.

## Introduction

Inflammatory bowel disease (IBD) is a highly heterogeneous group of diseases, with extensive variation in clinical manifestation and treatment response. One of the sources of this variation is differences in microbial composition between individuals. Although most of the studies on the role of the microbiota in the pathogenesis of IBD are focused on the bacterial component, there is increasing evidence highlighting the relevance of viral and fungal dysbiosis in the pathogenesis of IBD.^1-3^ An altered fungal microbiota composition has been shown to be a relevant feature also in other diseases affecting the digestive system like irritable bowel syndrome,^4^*Clostridioides difficile* infection,^5^ cirrhosis,^6^ or colorectal cancer.^7^

Fungi are ubiquitous microorganisms representing only a small fraction of the total human gut microbiome (0.1%) and most are unculturable.^8,9^ However, the implementation of high-throughput sequencing methods in recent years like 18S rDNA amplicon sequencing has allowed a much more extensive characterization of fungal gut microbiota diversity, underscoring a key role in gut homeostasis.^10,11^ Of note, fecal samples sequencing techniques detect both transient and gut resident fungi, which should be kept in mind when interpreting the results in the literature.

Several studies have demonstrated alterations in the fecal fungal microbiota composition in IBD, with significant variations in the *Basidiomycota/Ascomycota* ratio,^12^ an increase in *Candida*, and a decrease in *Saccharomyces*.^12-15^ In addition, elevated level of *S cerevisiae* mannan antibodies (ASCA) is a well-established biomarker for CD. ASCA positivity may predict the development of CD years prior to diagnosis, correlates with disease activity, and is more common in healthy relatives of patients with CD.^16-18^ Also, antifungal drugs, like fluconazole, show some promise in treating patients with UC,^19^ and the use of fungal probiotics—like *S boulardii*—prolongs clinical remission in CD.^20^ Finally, fungi can also influence the outcomes of therapy in IBD. A recent study has demonstrated the influence of the abundance of *Candida* in the results of fecal microbiota transplantation in UC patients^21^ and in the clinical response to infliximab,^22^ highlighting the relevance of the fungal mycobiome.

Here, we present an in-depth study of the fungal microbiota in a well-characterized, large cohort of Norwegian IBD patients. We show important differences in the mycobiome composition in IBD and within the different phenotypes. In addition, we report variations in composition with disease activity and explore the association fungal signatures with bad outcomes. The data presented in this study provide a rationale to expand our understanding of the role of the mycobiome in IBD pathogenesis, opening for interventional studies targeting specific fungal populations.

## Methods

### Clinical Material

Subjects were included prospectively among individuals referred for colonoscopy to the outpatient clinic at the Department of Gastroenterology and Hepatology, St. Olav’s University Hospital, Trondheim, Norway. The diagnosis of IBD was established according to standard clinical, radiological, endoscopic, and histological criteria. A total of 128 individuals were recruited. Of them, 23 subjects were excluded, leaving us with 105 individuals included in the final analysis: 84 IBD patients (46 UC and 38 CD) and 21 healthy control individuals. Exclusion criteria were: <18 years of age, uncertain IBD diagnosis or IBD–unclassified, recent colonoscopy in the previous 4 weeks, and use of antibiotics the last 3 months before inclusion. Healthy control individuals were healthy volunteers and patients with mild gastrointestinal symptoms in which standard diagnostic procedures revealed no significant disease. The number of subjects included in each analysis is indicated in the respective results section. Patient characteristics including age, sex, current medication, Montreal Classification phenotype, fecal calprotectin values (FC), neutrophil gelatinase-associated lipocalin (NGAL), and disease history are summarized in Table 1. An FC level >100 μg/mg was used as indicative of active disease.

**Table 1.** Subject characteristics.

Characteristic	Healthy control group (n = 21)	UC group (n 46)	CD group (n = 38)
Age, y	39 (22-71)	45 (18 -76)	39 (18 -72)
% Female	72.2	53.0	63.6
Montreal classification			
B1	—	—	5 (31.2)
B2	—	—	11 (68.7)
L1	—	—	6 (37.5)
L2/L3	—	—	10 (62.5)
E1	—	6 (31.6)	—
E2	—	6 (31.6)	—
E3	—	7 (36.8)	—
S0	—	6 (27.3)	—
S1	—	7 (31.8)	—
S2	—	9 (40.9)	—
S3	—	0 (0)	—
FC, µg/mg	<50 (—)	74 (<50-1663)	176 (<50-1569)
NGAL, mg/kg	0.28 (0-1.52)	2.14 (0.12-43.75)	2.12 (0.25-16.8)
Current medication			
5-ASA	—	29 (63.0)	5 (13.2)
Imurel	—	23(6.5)	5 (13.2)
Steroid	—	14 (30.4)	16 (42.1)
Anti-TNF	—	1 (2.2)	4 (10.5)

Values are median (range) or n (%), unless otherwise indicated.

The final patient cohort included samples from 105 participants. For 3 healthy control, 3 UC, and 5 CD individuals, the age and sex is unknown. FC/NGAL and Montreal classification was only noted for subsets of the samples, as noted in the respective results. Percentages reflect part of total known, excluding individuals where no FC/NGAL or Montreal classification is given.

Abbreviations: 5-ASA, mesalamine; CD, Crohn’s disease; FC, fecal calprotectin; NGAL, neutrophil gelatinase-associated lipocalin; TNF, tumor necrosis factor; UC, ulcerative colitis.

A subset of IBD patients was followed clinically for a period of 6 ± 2 years. Based on clinical observations, each individual’s disease course was designated as either complicated or uncomplicated based on the need of intensified medication and/or surgery. Patients who needed the addition of corticosteroids, an immunomodulator (azathioprine/6-mercaptopurine or methotrexate), an anti-tumor necrosis factor drug, or a surgical procedure related to the disease complications were classified as complicated. For CD patients, the group with a complicated disease course was further divided into 2 subsets, those needing surgery or not.

The study was approved by the Regional Committee for Medical and Health Research Ethics (reference numbers 5.2007.910 and 2013/212/REKmidt), and all subjects gave informed, written consent prior to inclusion.

### Fecal Samples

#### Stool Collection and DNA Extraction

Stool samples were collected, homogenized, and stored at − 80 °C for further analysis. From each sample, 200 mg of fecal matter was resuspended in 500 µL lyticase buffer (500 mM Tris, 1 mM EDTA, 0.2% 2-mercaptoethanol, 200 U Lyticase [Cat. No. L4025-25KU; Sigma-Aldrich), and incubated at 30 °C (30 minutes). Samples were centrifuged (1500 *g* for 5 minutes) and the supernatant was removed. After resuspension in 800 mL Stool DNA stabilizer (Cat. No. 1 038 111 100; B-Bridge International), the sample was transferred to Precellys tubes containing 100 µL, 0.1 mm and 300 µL, 0.5 mm glass beads. Lysis was performed through bead beating (2 rounds of 2 × 30 seconds, 6800 rpm; Precellys 24; Bertin Instruments), followed by incubation at 95 °C for 10 minutes. After 1 minute on ice, the lysed sample was centrifuged (13 000 *g* for 1 minute) and the supernatant was collected. DNA was isolated following the QIAamp DNA isolation protocol (Cat. No. 51 306; Qiagen). DNA concentration was assessed using spectrophotometry (NanoDrop; Thermo Fisher Scientific).

Analysis of NGAL and FC levels in fecal samples was performed as previously described.^23^ In brief, the samples were analyzed for NGAL using enzyme-linked immunosorbent assay (BioVendor R&D). FC was analyzed using enzyme-linked immunosorbent assay by Calpro AS. The samples were diluted 1:50 using Calpro EasyExtract and diluted according to manufacturer’s protocol.

#### ITS Sequencing

Fungal amplicons targeting the ITS1 sequence were prepared and sequenced as previously described.^24^ In brief, a 3 µL sample was used for amplification of the ITS1 sequence using Phusion DNA Polymerase (New England BioLabs). Polymerase chain reaction was run using the primers ITS1F (CTTGGTCATTTAGAGGAAGTAA) and ITS2 (GCTGCGTTCTTCATCGATGC) with added sample-specific barcodes. The resulting amplicons were used to generate Illumina TruSeq libraries, and subsequently sequenced on a MiSeq sequencer (Illumina), using paired-end sequencing. Raw data processing and de-multiplexing was performed using the software supplied with the MiSeq instrument according to manufacturer’s recommendations and exported as raw FASTQ files. FASTQ data were filtered on several criteria, including removing adapter sequences, any reads not including the ITS1F sequence, or any reads containing unknown bases. Reads were then trimmed using the split_libraries_fast.py-script in QIIME v1.6.^25^ Processed reads were aligned to the THF (Targeted host-associated fungi) database v1.5^24^ and a matrix in which each sample sequences were mapped to operational taxonomic unit (OTU) IDs was prepared for subsequent data analysis.

### Statistical Analysis

Data analysis was performed in R version 4.1.1 (R Foundation for Statistical Computing) and GraphPad Prism version 9.1.2 (GraphPad Software). Alpha- and beta-diversity calculations were performed on unfiltered and unnormalized data. Data import and abundance analysis was performed using the phyloseq tools.^26^ Three alpha-diversity measures were used: observed diversity, the Chao1 index, and the Shannon diversity index. Observed diversity is a count of richness of species, ie, the number of observed OTUs within each sample. The Chao1 index weighs the richness, allowing low abundance species to have greater influence on the score.^27^ The Shannon diversity index measure takes species richness into account, while at the same time adjusting for equitability in species abundance.^28^ Alpha-diversity measures were tested for significant differences between sample groups using nonparametric Kruskal-Wallis test with Dunn’s correction for multiple comparisons. Beta diversity plot is represented as a principal component analysis plot of the Bray-Curtis dissimilarity matrix (vegan, version 2.4-2).^29^Significance of beta diversity was assessed using permutational multivariate analysis of variance (adonis) using the Bray-Curtis dissimilarity matrix, with default parameters and 10 000 permutations. For evaluation of the difference in *Basidiomycota*/*Ascomycota* and % *Zygomycota,* outliers were masked from analysis using the GraphPad ROUT function, with Q = 0.1%. nonparametric Kruskal-Wallis test with Dunn’s correction for multiple comparisons was used to test for significant differences between sample groups. For the evaluation of global differences in species/genera abundances between the sample groups, the DeSeq2 package was used.^30^ Prior to DeSeq analysis, the dataset was filtered, removing all OTUs in which there were <5 samples showing more than 10 read counts. Furthermore, as mesalamine (5-ASA) use differed greatly between patient groups; 5-ASA status was included as control variable in all differential abundance calculations. DeSeq2 was also used to evaluate association between NGAL/FC levels and species/genera abundance, using log2-transformed NGAL/FC levels as continuous variables. In the case of FC, values <50 were not available, and were set to 20. All DeSeq2-calculated *P* values are adjusted for multiple comparisons using Benjamini-Hochberg correction.^31^

## Results

### Altered Fungal Microbiota Diversity in IBD

Because previous studies have shown changes in the gut fungal microbiota diversity in IBD,^12,14,32^ we wanted to establish whether this was the case in our study population including 84 IBD patients (46 UC and 38 CD) and 21 healthy control individuals (Table 1). We used FC level >100 μg/mg as a marker for active disease distinguishing 2 patient groups: active IBD (IBDa) (n = 46), of which 21 were active UC and 25 were active CD; and IBD in remission (n = 37), of which 24 were UC in remission and 13 were CD in remission. The alpha diversity within each patient group was assessed using 3 measures: (1) observed diversity, (2) Shannon diversity index, and (3) Chao1 index.

**Figure 1.**
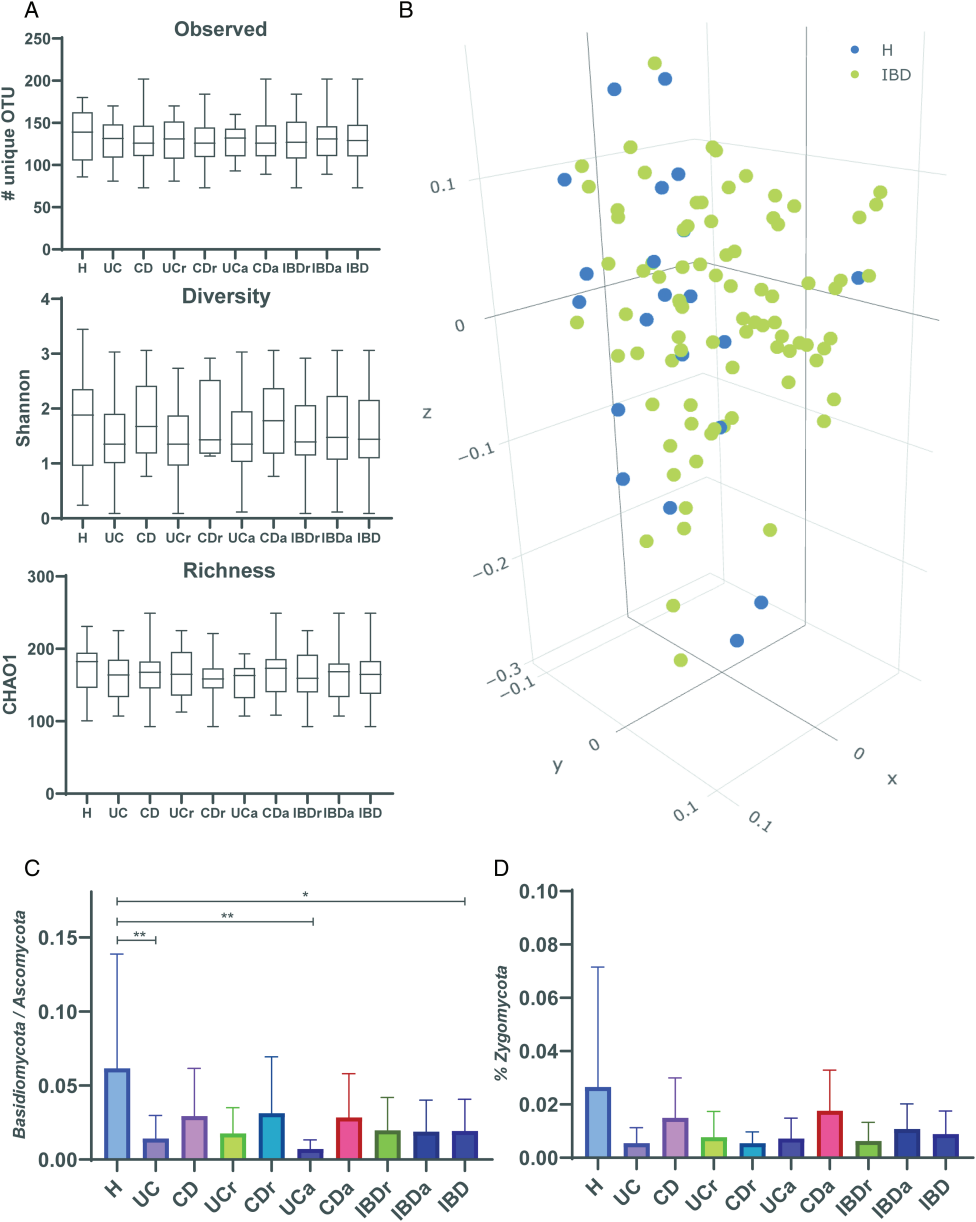
A, Alpha-diversity analysis of fungal species found in fecal samples from healthy control individuals (H) (n = 21), ulcerative colitis (UC) patients (n = 45), Crohn’s disease (CD) patients (n = 38), UC in remission (UCr) patients (n = 24), CD in remission (CDr) (n = 13) patients, active UC (UCa) patients (n = 21), active CD (CDa) patients (n = 25), UCr and CDr patients combined (IBDr) (n = 37), and UCa and CDa patients combined (IBDa) (n = 46) groups. B, Beta diversity of fungal species found in fecal samples from H (n = 21) and all IBD groups combined (IBD) (n = 84). There is a significant difference when comparing H and IBD groups (*P* = .003). C, *Basidiomycota/Ascomycota* ratio within each sample group. There is a significantly lower ratio within IBD sample groups than in fecal samples from healthy individuals. D, *Zygomycota* as percentage of whole. Although representing <1% of all species, the *Zygomycota* appear to represent a smaller proportion of all phyla from IBD patients compared with H. *P < .05, **P < .01.

Alpha diversity was reduced in both UC and CD compared with healthy control individuals, particularly in the case of UC patients vs healthy control individuals measured by the Shannon diversity index. However, none of the contrasts reached statistical significance (Figure 1A). When we compared the alpha diversity of the samples sorting by disease activity, we noted that patients with active CD presented a higher fungal diversity than CD patients in remission, although it was not significant. Interestingly, we found no difference in alpha diversity when comparing the total number of IBD patients in remission with active disease (Figure 1A).

Beta-diversity analysis of fungal species showed a significant difference when comparing IBD patients with healthy control individuals (IBD and healthy control groups: *P* = .003) (Figure 1B), and a close to significant difference when taking disease activity into consideration (active IBD, IBD in remission, and healthy control groups: *P* = .079). There was a significant difference in beta-diversity measures between the study groups (UC, CD, and healthy control groups: *P* = .005). If disease activity was considered, the distance measures were close to significantly different (active UC, active CD, UC in remission, CD in remission, and healthy control groups: *P* = .053).

### Changes in Fungal Microbiota Composition in IBD

The fungal phyla *Ascomycota* and *Basidiomycota* are the most prevalent in the human mycobiome.^4,12^ In our study population, *Ascomycota* was clearly predominant both in IBD patients and healthy control individuals (Figure 1C). We analyzed the differences in *Basidiomycota/Ascomycota* ratio, showing a significantly lower ratio in IBD patients, both in patients in remission and active patients. This reduction was especially significant in the case of UC patients *vs* control individuals and more marked in active UC (*P* = .0012) (Figure 1C). Similarly, the phyla Zygomycota appeared to be depleted in IBD patients compared with healthy control individuals, albeit significantly when compared with healthy control individuals (Figure 1D).

**Figure 2.**
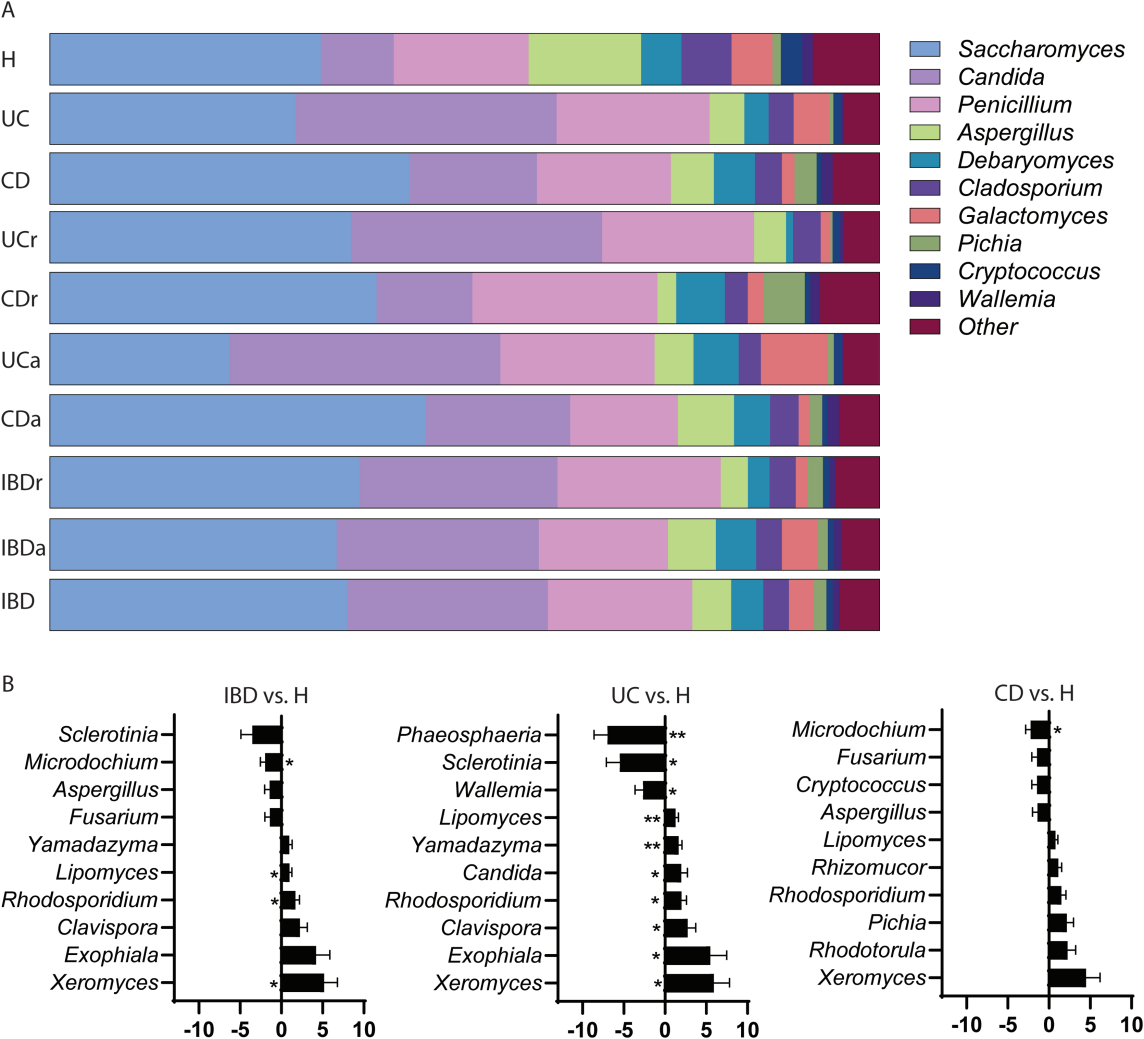
A, Illustration of the distribution of top 10 genera within all sample groups. B, Top 10 (adjusted *P* value) genera in the contrasts healthy control individuals (H) vs active inflammatory bowel disease (IBDa) patients (left), H vs active ulcerative colitis (UCa) patients (middle), and H vs active Crohn’s disease (CDa) patients (right). Of note, while several genera were significantly more abundant in UC vs H (*Xeromyces*, *Exophiala*, *Clavispora*, *Rhodosporidium*, *Candida*, *Yamadazyma*, and *Lipomyces*), we did not find any of those genera significantly overrepresented in CD samples alone. Moreover, only *Microdochium* was significantly depleted of in CD vs H. The number of samples in each sample group is the same as for Figure 1. *P < .05, **P < .01. CDr, Crohn’s disease in remission; IBDr, inflammatory bowel disease in remission; UCr, ulcerative colitis in remission.

The main differences at the genera level in the fungal mycobiome in IBD vs healthy control groups are depicted in Figure 2. The comparison between the top 10 most abundant genera within all sample groups shows that IBD samples present a relative increase in *Candida* abundance and a decrease in *Aspergillus*, *Debaromyces*, and *Cladosporium* (figure 2A).

To further characterize the mycobiome variations, we compared the top 10 genera that showed major differences in the contrast after adjusted *P* value. Patients with IBD showed a significant increase in *Xeromyces*, *Rhodosporium*, and *Lipomyces* and a significant reduction in *Microdochium* (*P* ≤ .05) (Figure 2B). We also found a significant increase of *Yadazyma* and *Lipomyces* in UC and a decrease in *Phaeosphaeria* (*P* ≤ .05), and a significant depletion of *Microdochium* in CD (*P* ≤ .05) (Figure 2B). Of note, although several genera were significantly more abundant in the UC vs healthy control groups, none of these genera were found to be significantly overrepresented in CD.

**Figure 3.**
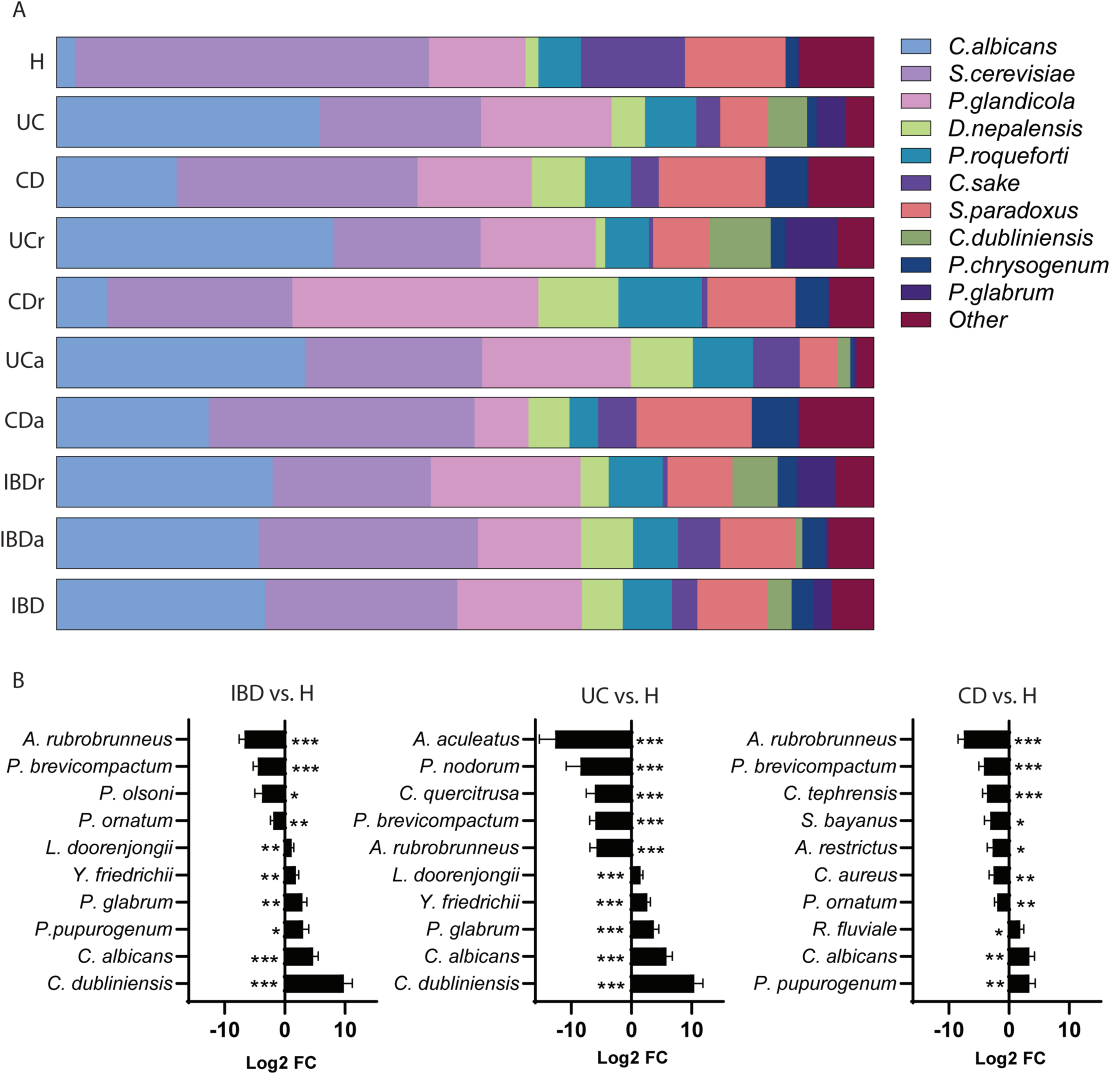
A, Illustration of the top 10 species across all sample groups. B, Top 10 (adjusted *P* value) Species in the contrasts inflammatory bowel disease (IBD) vs healthy control individuals (H) (left), ulcerative colitis (UC) vs H (middle), and Crohn’s disease (CD) vs H (right). The number of samples in each sample group is the same as for Figure 1. *P < .05, **P < .01, ***P < .001. CDa, active Crohn’s disease; CDr, Crohn’s disease in remission; IBDa, active inflammatory bowel disease; IBDr, inflammatory bowel disease in remission; UCa, active ulcerative colitis; UCr, ulcerative colitis in remission.

Then, we assessed the variations in fungal species between IBD patients and healthy control individuals (Figure 3). The characterization of the 10 most abundant species within all sample groups reveals a marked increase in *Ca albicans* and a decrease in *S cerevisiae* in IBD samples (Figure 3A).

The analysis of the top 10 species that showed major differences after adjusted p value showed a very significant increased abundance of *Ca dublinensis* and *Ca albicans* and decreased abundance of *A rubrobrunneus* and *Penicillium brevicompactum* in IBD vs healthy control groups (*P* ≤ .001) (Figure 3B). The abundance of *Ca dublinensis*, *Ca albicans*, *P glabrum*, *Yamadazyma friedrichii*, and *Lypomyces doorenjongii* was significantly increased in UC patients, while *A aculeatus, P nodorum*, *Ca quercitrusa*, *P brevicompactum*, and *A rubrobrunneus* were decreased (*P* ≤ .001) (Figure 3B). CD patients had a significant increase in *P pupurogenum* and *Ca albicans* (*P* ≤ .01) and a depletion of *A rubrobrunneus, P brevicompactum*, *and Cryptococcus tepherensis* (*P* ≤ .001).

### Variations of Fungal Composition in the Different Disease Phenotypes

**Figure 4.**
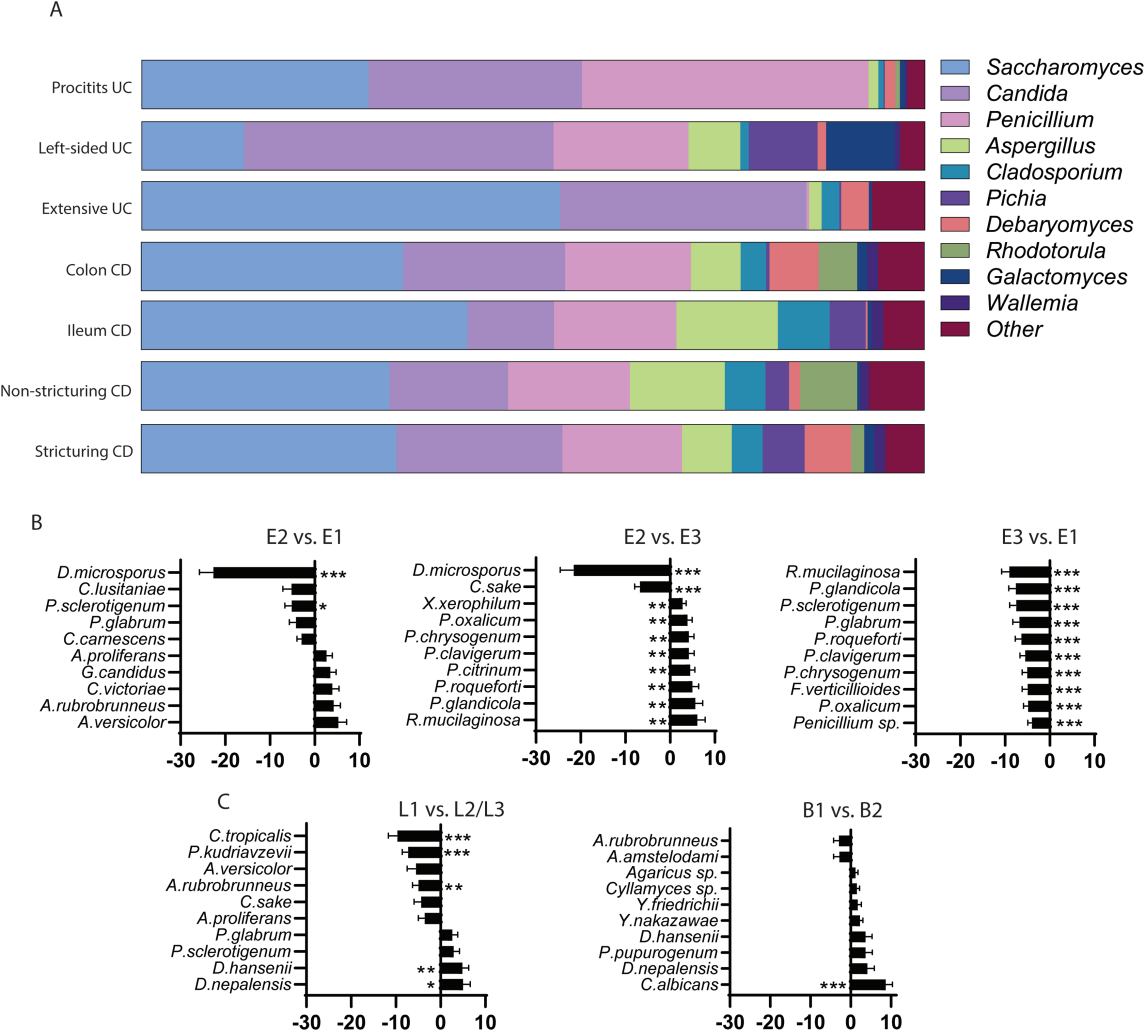
Montreal classification and mycobiome. The figure shows the variation of the fungal microbiome in the different ulcerative colitis (UC) and Crohn’s disease (CD) clinical phenotypes. A, Illustration of the distribution of top 10 genera within groups of patients based on Montreal classification. Colon CD = Montreal CD L1 (n = 6); ileum CD = Montreal CD L2/L3 (n = 10); nonstricturing CD = Montreal CD B1 (n = 5); fibrostenosing CD = Montreal CD B2 (n = 11); proctitis UC = Montreal UC E1 (n = 5); left-sided UC = Montreal E2 (n = 6); extensive UC = Montreal UC E3 (n = 7). B, Top 10 (adjusted *P* value) species within the contrasts left-sided UC vs proctitis (left), left-sided UC vs extensive UC (middle), and extensive UC vs proctitis (right). C, Top 10 (adjusted *P* value) species within the contrast colonic CD vs ileal CD (left), and stricturing CD vs nonstricturing CD (right). *P < .05, **P < .01, ***P < .001.

IBD is a heterogeneous entity encompassing different disease phenotypes. The variations of the mycobiome in the different Montreal classification scenarios has been poorly characterized. Thus, we studied the variations in fecal fungal composition regarding localization and disease behavior (Figure 4). The relative abundance of the 10 most abundant genera in the 3 main UC phenotypes (proctitis: E1; left-sided colitis: E2; extensive colitis: E3) is shown in Figure 4A. Patients with proctitis present the highest abundance of *Penicillium*, which decreases in patients with left-sided colitis and virtually disappears in patients with extensive colitis. Of note, *Pichia* was overrepresented in patients with left-side colitis in comparison with proctitis, and we did not find any variations of *Candida* within UC phenotypes (Figure 4A).

The analysis of the top 10 species showed a significant increased abundance of *Rhodotorula mucilaginosa*, *Xerochrisium xerophilum*, and several *Penicillium* species (*P glandicola, P roqueforti, P citrinum, P clavigerum, P chrysogenum*, and *P oxalicum*) (*P* ≤ .01); and a decrease in *Ca sake* and *D microsporus* (*P* ≤ .001) in left-sided colitis vs extensive colitis samples (Figure 4B). Interestingly, *D microsporus* was also markedly depleted in left-sided colitis compared with proctitis. We also found a very significant decrease in the abundance of some *Penicillium* species and *R. mucilaginosa* in patients with extended colitis vs proctitis (Figure 4B).

Recent evidence points to the importance of differentiating 2 major CD phenotypes beyond Montreal classification. Several groups have suggested to split CD into 2 distinctive groups: ileum dominant (pure ileal and ileocolonic) and isolated colonic disease, due to their genetic and immunological differences and their different response to treatment.^33,34^ Thus, it was interesting to study the variations of the mycobiome in the different CD locations. The differences in the most abundant fungi genera for these categories are plotted in Figure 4A. Notably, we found a relative increase in *Candida* and *Debaromyces* in colonic forms of CD, while *Aspergillus* and *Pichia* were increased ileal CD. At the species level, CD patients with pure ileal (L1) localization showed a significant decrease in *Ca tropicalis* and *P kudriavzevii* (*P* ≤ .001) and an increase in D *hansenii* (*P* ≤ .01) (Figure 4C).

Data on the changes in the mycobiome within the different behavior CD phenotypes (B, Montreal classification) are scarce. In our samples, patients with stenosing behavior (B2) showed a very marked increase in *Ca albicans* compared with patients with the inflammatory phenotype (B1) (*P* ≤ .001). In fact, that was the only significant variation when the fungal composition was compared between colonic and ileal CD (Figure 4C).

### Correlation Between Fungal Populations and Activity of the Disease

**Figure 5.**
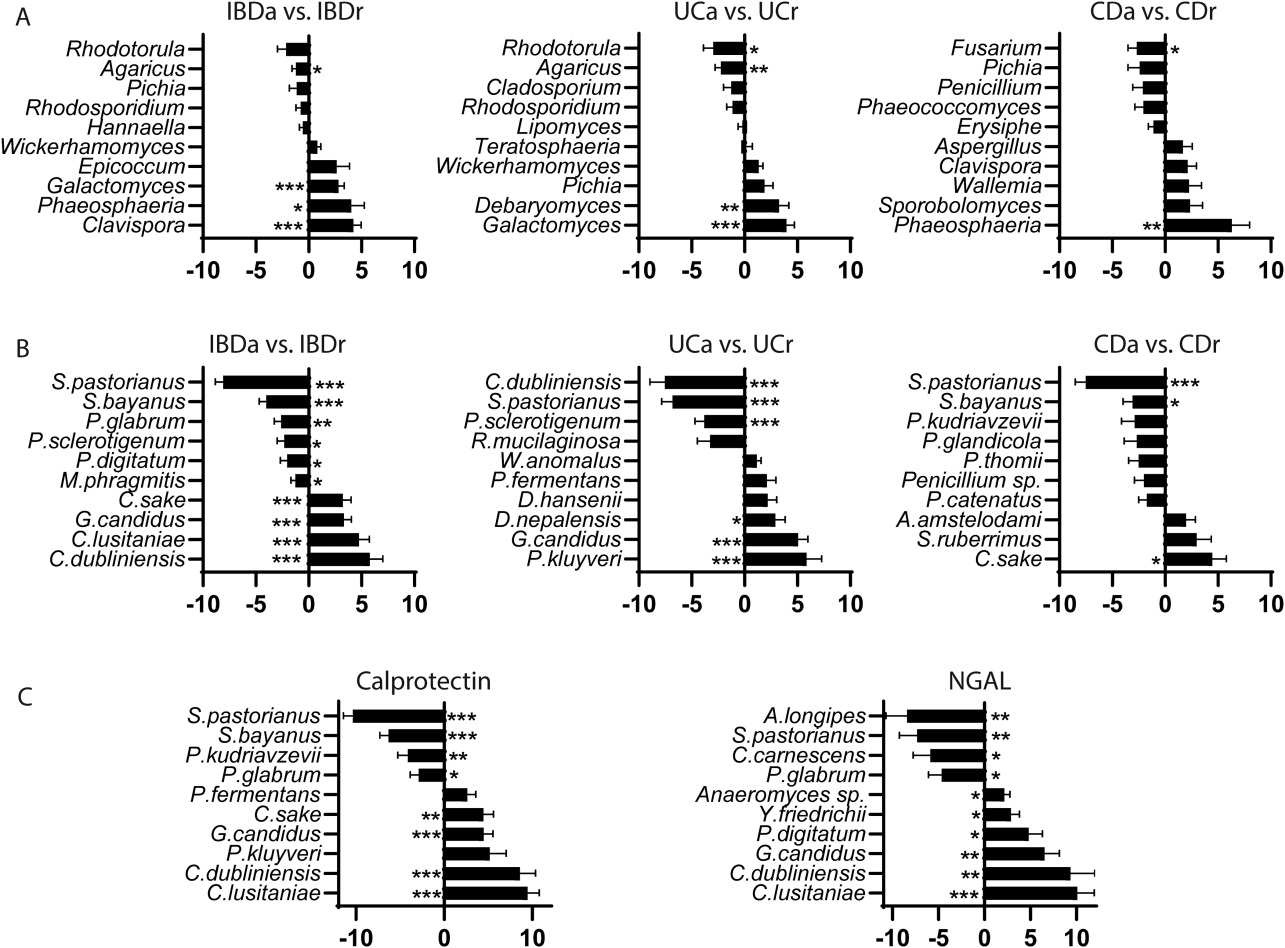
Disease activity and mycobiome: A and B, Fecal calprotectin (FC) levels were available for study in 83 inflammatory bowel disease (IBD) patients. An FC level >100 μg/mg was used as indicative of active disease. Using this, each of the sample groups (ulcerative colitis [UC], n = 45; Crohn’s disease [CD], n = 38; IBD, combination of UC and CD patients) were divided into active and remission. Plots show top 10 (adjusted *P* value) genera (A) and species (B) for the contrasts active IBD (IBDa) (n = 46) vs IBD in remission (IBDr) (n = 37) (left), active UC (UCa) (n = 21) vs UC in remission (UCr) (n = 24) (middle), and active CD (CDa) (n = 25) vs CD in remission (CDr) (n = 13) (right). C, We analyzed the association between FC and neutrophil gelatinase-associated lipocalin (NGAL) levels with species abundance within the IBD cohort. C, Top 10 (adjusted *P* value species when associating with FC (n = 83) (left) and NGAL (n = 82) (right) levels. *P < .05, **P < .01, ***P < .001.

To help understanding the possible association of fungal composition and inflammation, we compared the mycobiome of patients with active disease with those in remission using a cutoff of FC level of >100 μg/mg as indicative of activity. Active IBD patients showed increased abundance of *Clavispora* and *Galactomyces* (*P* ≤ .001), UC patients presented increased *Galactomyces* (*P* ≤ .001) and *Debaromyces* (*P* ≤ .01), and CD patients had increased abundance of *Phaeosphaeria* (*P* ≤ .01) and a depletion in *Fusarium* (*P* ≤ .05) (Figure 5A). Moreover, active IBD patients showed an increase in 3 different *Candida* species (*Ca dublinensis*, *Ca luistaniae*, and *Ca sake*) and *Galactomyces candidus*, while *S pastorianus* and *S bayanus* were depleted (*P* ≤ .001) (Figure 5B). In active UC, a significant increase in *P kluyveri* and *G candidus* was noted (*P* ≤ .001), while *Ca dublinensis*, *S pastorianus*, and *P sclerotigenum* were less abundant (*P* ≤ .001) than in UC patients in remission. However, CD active patients have an increased abundance of *Ca sake* (*P* ≤ .05) and a very marked depletion of *S pastorianus* compared with CD in remission (*P* ≤ .001) (Figure 5B).

We analyzed the association between the disease activity biomarkers FC and NGAL levels with fungal species composition within the IBD cohort. Interestingly, an increase in *Ca dublinensis* and *Ca luistaniae* and a decrease in *S pastorianus* is associated with increased levels of both FC and NGAL (Figure 5C).

### Fungal Composition and Disease Course

**Figure 6.**
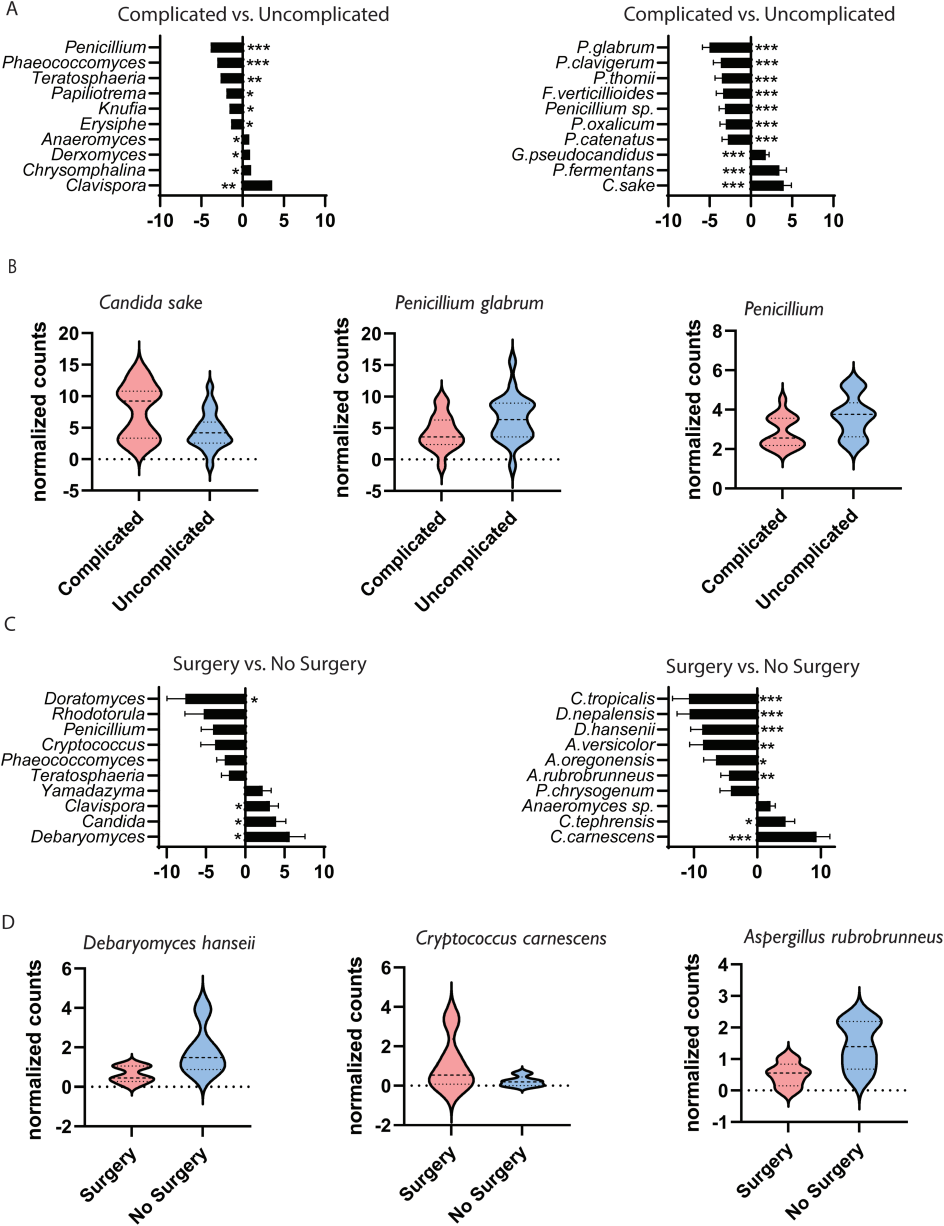
Mycobial diversity and disease course. A subset of inflammatory bowel disease patients was followed clinically in a period of 4 to 8 years. Based on clinical observations, each individual’s disease course was designated as either complicated or uncomplicated based on the need of intensified medication and/or surgery. For Crohn’s disease, the group with a complicated disease course were further divided into subsets needing surgery or not. A, Top 10 (adjusted *P* value) genera (left) and species (right) in the contrast complicated (n = 17) vs uncomplicated (n = 23). B, Plot of individual species and genus showing significant difference between the groups complicated (n = 17) and uncomplicated (n = 23). C, Top 10 (adjusted *P* value) genera (left) and species (right) in the contrast Crohn’s disease patients needing surgery (n = 5) vs no surgery (n = 5) (right). D, Plot of individual species showing significant difference between the groups surgery (n = 5) and no surgery (n = 5). *P < .05, **P < .01, ***P < .001.

A characterization of the fecal mycobiome in relation to IBD outcomes has, to our best knowledge, not been performed to date. Thus, we proceeded to analyze the association of fungal populations in patients with disease outcomes. A subset of IBD patients (n = 40) was followed clinically for a period of 6 ± 2 years and each individual’s disease course was designated as either complicated or uncomplicated based on the need of intensified medical treatment and/or surgery (Figure 6). Patients with complicated disease had significantly more *Clavispora* (*P* ≤ .01) and less *Penicillium* and *Phaeococcomyces* (*P* ≤ .001) than those with an uncomplicated course. At the species level, a complicated course was associated with more abundant *Ca sake*, *P fermentans*, and *G pseudocandidus* and a reduction in several *Penicllium* species (*P glabrum*, *P clavigerum*, *Pthomii*, *P oxalicum*, and *P catenatum*) (*P* ≤ .001) (Figure 6A and 6B).

There is increasing interest in characterizing CD patients that are at high risk of needing surgery early in the disease course to implement more potent treatment strategies and closer monitorization. Thus, we studied of the association of fungal species with the need for surgery in CD patients during follow (shown in Figure 6C). We found a strong association between a decreased abundance of *Ca tropicalis*, *D nepalensis*, and *D hansenii* and the need for surgery in CD (*P* ≤ .001). Increased abundance of *Cr carnescens* was also markedly associated with the need of surgery during follow-up (*P* ≤ .001). Plots of individual species showing highly significant association with either the disease course groups or CD patients needing surgery are shown in Figure 6C.

## Discussion

This study presents an in-depth characterization of the fecal fungal microbiome in patients with IBD and its correlation with different disease phenotypes and clinical activity providing new evidence on the role of the mycobiome in both UC and CD. We also describe, for the first time to our knowledge, a significant association of fungal signatures preceding worse outcomes after clinical follow-up.

There is a wide variation in fungal diversity in IBD in the studies published to date. Our study shows a decreased alpha diversity in IBD patients, both in UC and CD samples, although without reaching statistical significance. We did not find any significant differences in diversity between UC and CD, although a more marked decrease in diversity was noted in UC. These results are in line with previous studies showing a decreased fungal diversity in IBD.^12-14,16,35^ Beta-diversity analysis showed a clustering of IBD samples and control individuals, which was significant, a finding that is consistent with previous studies.^12,32^

A novel finding in our work is the significant depletion of *Zygomycota* in IBD patients, independently of disease activity, which was not previously described in the literature. *Zygomycota* are characterized by the formation of zygospores and represent a small proportion of the human mycobiome.^36^ Interestingly, depletion in *Zygomycota* has recently been described in obese patients, and the depletion of *Mucor* genus was reversible upon weight loss.^37^ The implications of the depletion of *Zygomycota* in IBD deserve further study.

We identified 5 genera, *Saccharomyces*, *Penicillium*, *Debaromyces*, *Aspergillus*, and *Candida*, as the most abundant both in healthy control individuals and IBD patients, which is concordant with small variations with other European cohorts.^12,32,38^ IBD samples present a relative increase abundance of *Candida*, while *Aspergillus*, *Debaromyces*, and *Cladosporium* were reduced. However, only *Xeromyces*, *Rhodosporium*, and *Lipomyces* were significantly more abundant in IBD. Interestingly, the mycobiome at the genus level was different between UC and CD, highlighting the microbiological differences between them, as previously reported in gut bacteria.^35^

Our study reveals that IBD patients present an increased abundance of *Ca albicans* and *Ca dublinensis*. An overrepresentation of *Candida* has been consistently reported in the literature both in adult^15,39,40^ and pediatric IBD.^14^ A recent Chinese study also found an expansion of *Candida* in CD, but only in the active group, suggesting a participation in inflammation.^39^ In line with this, a deleterious role of *Candida* expansion has recently been described in clinical studies of patients with IBD. High *Candida* abundance was associated with a clinical response in UC patients treated with fecal microbiota transplantation with a decrease in *Candida* in those patients who responded.^21^ Also, a recent study demonstrated high abundance of *Candida* in nonresponders to infliximab, highlighting its potential proinflammatory effect.^22^

In particular, a possible role of *Ca albicans* in the pathogenesis of IBD was previously suggested in several animal models of colitis^41,42^ as well as in clinical studies. In fact, CD patients and their first-degree healthy relatives have been shown to be more frequently and heavily colonized by *Ca albicans* than control individuals, and ASCA levels correlate with *Ca albicans* colonization in relatives.^15^ Interestingly, Sokol et al^12^ also reported an increase of the relative abundance of *Ca albicans* during flares. However, although *Ca albicans* was significantly more abundant both in UC and CD in our samples, it was not significantly overrepresented in active patients and its abundance did not correlate with inflammation markers.

On the other hand, *Ca dublinensis* abundance was significantly higher only in patients with CD, it was clearly overrepresented in active IBD patients, and correlated strongly with serological markers of inflammation in our patients. *Ca dubliniensis* is phylogenetically closely related to *Ca albicans* and was originally described to cause oral candidiasis in HIV-infected individuals and AIDS patients.^43,44^ The role of *Ca dubliniensis* as a potential opportunistic pathogen in CD, in which several innate immunity defense mechanisms are impaired,^45,46^ deserves further investigation.

Both UC and CD patients were markedly depleted in *Aspergillus*, a finding that is consistent with previous reports.^47^*A aculeatus* was less abundant in UC, while *A rubrobrunneus* (*A ruber*) was significantly depleted in both UC and CD. Interestingly, *P brevicompactum* was also very significantly depleted in UC and CD. *P brevicompactum* is a filamentous fungus widely distributed throughout the natural world and is also used in the production of Mycophenolic acid,^48,49^ a compound that has anti-inflammatory effects in IBD (mycophenolate mofetil).^50^ Two recent studies have also shown a reduction in *Penicillium* in European and Chinese patients with IBD,^12,47^ although some other studies found no significant differences.^51^ One of the most striking findings in our study focusing on IBD phenotypes was the decreasing abundance of *Penicillium* from UC proctitis to extensive colitis, in which it virtually disappears. This was corroborated at species level (*P glandicola*, *P roqueforti*, *P citrinum*, *P clavigerum*, *P chrysogenum*, and *P oxalicum)*. This is, to our best knowledge, the first time that an inverse correlation of *Penicillium* and disease extension is described in UC. In line with this, a potential beneficial effect of this fungus in IBD was previously suggested by Sokol et al,^12^ who described a decrease in *Penicillium* in IBD patients, more marked in active patients, as well as a recent report showing a decrease of *Penicillium* in CD.^47^ In contrast, although *Candida* is overrepresented in UC samples, there was no difference in abundance between the 3 different UC phenotypes in our study (E1, E2, and E3).

Recent findings highlight the genetical, immunological, and clinical differences between ileal and colonic CD.^33,34^ However, little is known about the changes of the mycobiome composition in the different CD locations. In our study, patients with isolated ileal CD showed a marked increased abundance in *D hansenii*, a species previously related to CD pathogenesis. A recent study by Jain et al^52^ demonstrated that *D hansenii* is enriched in inflamed ileal biopsies of patients with CD and impairs mucosal healing. In addition, a recent report found an enrichment in *Debaromyces* in patients with UC.^39^ Our findings point to a specific role of *D hansenii* in ileal CD and suggest this fungus as a potential therapeutic target in this subset of patients.

Remarkably, we also found a clear depletion of *Ca tropicalis* in patients with isolated ileal CD compared with patients with colonic involvement (L2/L3). Hoarau et al^13^ found that patients with CD have much higher abundance of *Ca tropicalis* compared with their first-degree relatives without CD, and a recent study showed that *Ca tropicalis* induces dysbiosis leading to increased intestinal permeability and induces T helper 1/T helper 17 responses in mice.^40^ The specific pathogenic role of *Ca tropicalis* in CD need to be confirmed in larger cohorts.

We found a different mycobiome composition in patients with inflammatory type of CD (B1) versus stenosing CD (B2). To our knowledge, this is the first time that the fungal microbiome variations are studied in the different behavior (B) phenotypes of the Montreal classification. In our study, *Ca albicans* is largely overrepresented in inflammatory (B1) vs stricturing (B2) phenotypes, and it is actually the only species showing significant differences. An increased abundance of *Ca albicans* in inflammatory CD (B1) is consistent with its proinflammatory effects described in several studies.^12,42,53^ Additional factors like differences in gut transit time, variations in oxygen concentration in inflamed areas, or different dietary patterns in patients with stenosing CD could also contribute to these differences.

We then addressed the influence of disease activity on mycobiome composition to understand the clinical role of the intestinal fungi in human IBD. Active IBD patients showed an increase in 3 different *Candida* species (*Ca dublinensis*, *Ca luistaniae*, and *Ca sake*) with a depletion in *Saccharomyces* in line with previous studies.^12,47,53^ This pattern was also followed when we studied the correlation of the different species with FC and NGAL.

Several studies suggest a protective role of *Saccharomyces* in IBD.^12,16,41,54,55^*S cerevisiae* has demonstrated anti-inflammatory effects against colitis in murine models,^55^ and it has been shown that IBD patients and patients in flare present significantly less *S cerevisiae*.^12,16^ Moreover, mucosal-associated fungal studies have demonstrated an increase of *S cerevisiae* in noninflamed mucosa in CD patients,^54^ in agreement with our findings. Specifically, our results suggest a protective role of *S pastorianus* in IBD, since it was significantly depleted both in UC and CD patients in activity. *S pastorianus* is a hybrid yeast used for the production of lager beer.^56^ Interestingly, a combination of yeasts that included *S pastorianus* has showed promising anti-inflammatory results in a mice model of colitis^57^ and could represent an interesting therapeutic approach.

A study of the fecal mycobiome in relation to disease outcomes in IBD has, to our best knowledge, not been systematically performed to date. Thus, we followed a cohort of patients clinically and classified them as complicated or uncomplicated according to disease course.

Our analysis revealed that patients with complicated disease had significantly more *Clavispora* and less *Penicillium* abundance. At species level, *Ca sake* was most prevalent in patients developing complications during follow-up. *Ca sake* has a wide environmental distribution and is commonly found in human feces.^58^ Typically, *Ca sake* is present in food like grape juice, sauerkraut, and frozen salmon and does not grow at 37 °C.^59^ Because we did not record the dietary habits of IBD patients in our study, the association of its presence with aliment consumption needs to be elucidated in dedicated studies. Notably, although *Ca sake* rarely causes infections, it has been associated with severe cases of endocarditis, peritonitis, and bloodstream infections.^60^ Whether the colonization of *Ca sake* has a proinflammatory effect per se needs to be elucidated with mechanistic studies. In addition, our study found that a wide range of *Penicillium* species (*P glabrum*, *P clavigerum*, *P thomii*, *P oxalicum*, and *P catenatum*) were significantly depleted in patients with complicated disease, suggesting a potentially beneficial role, as previously pointed out in other cohorts.^12,47^

Despite the introduction of biologics, a significant proportion of CD patients still need surgery for disease complications over the years,^61^ which makes the identification of biological signatures early on a priority. Previously, certain bacterial populations have been linked to the risk of postoperative recurrence,^62^ but correlation between fungal composition and need for surgery in CD remains largely unexplored. We found a strong association between *Cr carnescens* abundance and the need for surgery. *Ca carnescens* has been classified as phylogenetic group II in the *Cr laurentii* complex and has been detected in wine grapes.^63,64^ To date, no studies have found a role of *Ca carnescens* in gut inflammation or IBD. Thus, the possible deleterious role of *Ca carnescens* in CD deserves further study. Surprisingly, we found a marked depletion of *Ca tropicalis* in those CD patients requiring surgery during disease course. This finding contrasts with a previous study suggesting a proinflammatory role of *Ca tropicalis* in CD.^14^ A possible explanation for this discrepancy could be the decreased abundance of *Ca tropicalis* found specifically in CD patients with isolated ileal affection in our study, which is a subgroup with increased risk to undergo surgery over time.

The present study has limitations. It includes only 1 fecal sample of IBD patients commonly seen in a tertiary hospital setting and with heterogeneous phenotypes and disease course. In addition, studying fecal samples includes by definition the fungi that are transient and thus highly influenced by dietary patterns, which was not systematically reported in our study. The patients included were not treatment naïve. Even though individuals receiving antibiotics were excluded, and all analyses were performed while correcting for 5-ASA use, there is a potential for some of our observation to be influenced by pharmacological treatment. Last, our work does not include a bacterial microbiome analysis, which could be interesting to study the bacterial-fungal networks and interactions to generate hypothesis.

Further studies are needed to describe in detail what a normal mycobiome looks like in populations with different genetic backgrounds, socioeconomic and environmental exposure, and dietary patterns. This can lead to the implementation of tailored microbiome-targeted therapies (probiotics, fecal microbiota transplantation, antifungal therapy, etc.) to restore balance and improve gut homeostasis. Furthermore, the identification of specific fungal signatures in IBD patients should lead to mechanistic studies that demonstrate causation beyond association. Several attempts in that direction are underway.^52,65^

## Conclusions

Our study shows relevant changes in the fecal mycobiome composition of Nordic patients with IBD, a poorly studied population to date. Patients with IBD show fungal dysbiosis, with significant differences in beta diversity and, in particular, genus and species that correlate with the activity of the disease. A systematic study of the changes in the mycobiome in the different disease phenotypes is an original addition of this study and may help to explore more precise microbiome-targeted strategies. The present work also shows for the first time an association of fungal signatures with worse disease outcomes and the need for surgery pointing to the potential use of mycobiome analysis as a tool for precision medicine in IBD. A better characterization of fungal populations in IBD would allow to elucidate the specific role of fungi in the pathogenesis of IBD. The study of the role of the fungal microbiome should help us to translate into better diagnostic and therapeutic strategies that improve the quality of life of patients with IBD.

## Data Availability

The sequencing data underlying this article are available at NCBI [https://submit.ncbi.nlm.nih.gov/subs/bioproject/SUB11802592/overview] with access number [BioProject ID: PRJNA911974].
