# Correction to: Fungal Microbiota Composition in Inflammatory Bowel Disease Patients: Characterization in Different Phenotypes and Correlation With Clinical Activity and Disease Course

**DOI:** 10.1093/ibd/izae070

**Published:** 2024-03-14

This is a correction to: Ignacio Catalán-Serra, Silje Thorsvik, Vidar Beisvag, Torunn Bruland, David Underhill, Arne Kristian Sandvik, Atle van Beelen Granlund, Fungal Microbiota Composition in Inflammatory Bowel Disease Patients: Characterization in Different Phenotypes and Correlation With Clinical Activity and Disease Course, *Inflammatory Bowel Diseases*, 2023;, izad289, https://doi.org/10.1093/ibd/izad289

The following change has been made to the original publication of this manuscript.

In the **Abstract**, under **Results**, the first sentence now reads: “We found lower *Basidiomycota/Ascomycota* ratio in IBD” instead of: “We found lower *Ascomycota/Basidiomycota* ratio in IBD”.

The emendation has been made to the article.

